# Features and drivers for energy-related carbon emissions in mega city: The case of Guangzhou, China based on an extended LMDI model

**DOI:** 10.1371/journal.pone.0210430

**Published:** 2019-02-11

**Authors:** Changjian Wang, Kangmin Wu, Xinlin Zhang, Fei Wang, Hongou Zhang, Yuyao Ye, Qitao Wu, Gengzhi Huang, Yang Wang, Bin Wen

**Affiliations:** 1 Key Laboratory of Guangdong for Utilization of Remote Sensing and Geographical Information System, Guangdong Open Laboratory of Geospatial Information Technology and Application, Guangzhou Institute of Geography, Guangzhou, China; 2 School of Geography, Geomatics and Planning, Jiangsu Normal University, Xuzhou, China; 3 College of Geography Science and Tourism, Xinjiang Normal University, Urumqi, China; 4 College of Economic and Management, Huanghuai University, Zhumadian, China; Universita degli Studi di Foggia, ITALY

## Abstract

Based on the apparent energy consumption data, a systematic and comprehensive city-level total carbon accounting approach was established and applied in Guangzhou, China. A newly extended LMDI method based on the Kaya identity was adopted to examine the main drivers for the carbon emissions increments both at the industrial sector and the residential sector. Research results are listed as follow: (1) Carbon emissions embodied in the imported electricity played a significant important role in emissions mitigation in Guangzhou. (2) The influences and impacts of various driving factors on industrial and residential carbon emissions are different in the three different development periods, namely, the 10^th^ five-year plan period (2003–2005), the 11^th^ five-year plan period (2005–2010), and the 12^th^ five-year plan period (2010–2013). The main reasons underlying these influencing mechanisms were different policy measures announced by the central and local government during the different five-year plan periods. (3) The affluence effect (***g-effect***) was the dominant positive effect in driving emissions increase, while the energy intensity effect of production (***e-effect-Production***), the economic structure effect (***s-effect***) and the carbon intensity effect of production (***f-effect-Production***) were the main contributing factors suppressing emissions growth at the industrial sector. (4) The affluence effect of urban (***g-effect-AUI***) was the most dominant positive driving factor on emissions increment, while the energy intensity effect of urban (***e-effect-Urban***) played the most important role in curbing emissions growth at the residential sector.

## Introduction

With anthropogenic releases of carbon emissions contributing toward climate change primarily characterized by global warming, many policymakers and scientific-researchers are seeking measures to mitigation [1–5]. As a result of the rapid urbanization, the world’s urban population grew from 2259.24 million in 1990 to 3784.39 million in 2013 (52.74% of the world’s population) [6]. Today, more than half of the world’s population lives in cities. International Energy Agency (IEA) estimated that energy-related carbon emissions in cities would grow by 1.8% annual, with the proportion of carbon emissions in cities rising from 71% to 76% during 2006 to 2030[7]. Therefore, cities are the main sources of carbon emissions throughout the world. Cities are also the major components in the implementation of carbon emissions mitigation measures [8]. City-based climate works are becoming popular, such as the *C40 Cities Climate Leadership Group* [9]. Examining and understanding the features and drivers of carbon emissions in cities is considered a fundamental step for implementing “low carbon city” strategies and actions.

With the rapid economic growth, China’s urban population grew from 300.17 million in 1990 (26.44% of the national total population) to 721.69 million in 2013 (53.17% of the national total population) [6]. Accompanying its rapid urbanization and industrialization, energy consumption and carbon emissions are increasing accordingly, aiming to meet the increased demands for infrastructure and services [10, 11]. China’s total CO_2_ emission has increased rapidly from 4085.6 million tonnes in 2003 to 9534.2 million tonnes in 2013 [12]. China has become one of the world’s largest energy consumer and carbon emitter [13, 14], accounting for one-quarter of all global carbon emissions [15]. Tackling climate change and addressing carbon mitigation, China promised to stabilize carbon emissions by 2030 in the “2014 U.S.-China Joint Announcement on Climate Change” [16, 17]. China proposed in the 13th Five-Year Plan (2016–2020) that, per unit of gross domestic product (GDP), energy consumption and CO_2_ emission should be reduced by 15% and 18% in 2020, respectively, compared with the 2015 levels [18]. Among carbon emissions sources in China, approximately 85% of emissions are contributed by energy consumption in cities, which is much higher than that experienced in the U.S. (80%) or in the EU (69%) [19, 20]. Consequently, it is particularly important to understand China’s carbon emissions in cities, and provide city-based evidence for energy conservation and emission mitigation actions.

Under such a circumstance, a vast body of studies has been conducted by domestic and foreign scholars to uncover the main drivers for China’s increasing carbon emissions in cities. As mentioned in Table 1, current research on carbon emissions in cities are mainly focusing on two research strands. The first strand of research focuses on the city-level total carbon accounting. Accurate accounting is crucial for efforts to curb the carbon rise. Previous studies on city-level total carbon accounting can be classified into 3 scopes [21–23]: Scope 1 –the direct carbon emissions produced by fossil fuel combustion and industrial process occurring within the city boundary; Scope 2 –the indirect carbon emissions related to imported electricity, steam and heating out of the city boundary; Scope 3 –the transferred carbon emissions embodied in imported/exported products and services out of the city boundary. Scope 1 and Scope 2 are calculated mainly based on the “Urban Emissions Inventory” as the product of activity sector and fuel data bases and appropriate emission factors. Especially, the emission factors for calculating Scope 2 emissions needs to be considered by the corresponding regional power supply system [21]. Scope 3 is calculated from the transferred carbon emissions embodied in imported/exported products and services in order to tackle the issue of emission leakage, which is mainly based on the environmentally extended Input-output (IO) models and hybrid Life Cycle Assessment (LCA) methodology. Thus the sources of data for accounting emissions embodied in imports and exports are critical for Scope 3 [24]. However, current emissions reduction targets are mainly based on Scope 1 emissions and without fully considering Scope 2 and Scope 3 emissions. In fact, findings of previous studies indicated that developed regions outsourced more emissions to other regions at the global [25], national [26], and local [27] scales. Therefore, comprehensive inventory and specific boundary are the basis for city-level carbon emissions accounting, as well as local emission mitigation strategies [28].

**Table 1:** Summary of selected China’s carbon emission studies in cities.

Authors	Time period	Methodology	Cities
		**City-level total carbon accounting**	
Sugar et al.[29]	2006	Urban Emissions Inventory	Beijing, Shanghai, and Tianjin
Wang et al.[30]	2002–2008	Urban Emissions Inventory	Shanghai
Li et al.[31]	2000–2010	Urban Emissions Inventory	Macao
Bi et al.[32]	2002–2009	Urban Emissions Inventory	Nanjing
Xi et al.[33]	2007	Urban Emissions Inventory	Shenyang
Zhao et al.[34]	2000–2009	Urban Carbon Flow	Nanjing
Liang et al.[35]	2005	IO	Suzhou
Li et al.[36]	2005–2009	IO	Macao
Chen et al.[37]	2007	IO	Beijing
Vause et al.[38]	2007	IO	Xiamen
Lin et al.[39]	2009	IO-LCA	Xiamen
Mi et al.[40]	2007	IO	Beijing, Shanghai, Tianjin, Chongqing, Dalian, Harbin, Ningbo, Qingdao, Shenyang, Shijiazhuang, Tangshan, and Xian
		**City-level driving factors and influencing mechanisms**	
Wang et al.[41]	1997–2010	STIRPAT	Beijing
Wang et al.[42]	2000–2010	LMDI	Beijing
Mi et al.[43]	2010	IO	Beijing
Wang et al.[44]	1997–2010	SDA	Beijing
Tian et al.[45]	1995–2007	SDA	Beijing
Wang et al.[46]	1996–2010	LMDI and Tapio index	Beijing, Tianjin
Wang et al.[47]	1998–2009	STIRPAT	Shanghai
Shao et al.[48]	1994–2009	STIRPAT	Shanghai
Zhao et al.[49]	1996–2007	LMDI	Shanghai
Shao et al.[50]	1994–2011	LMDI	Shanghai
Kang et al.[51]	2001–2009	LMDI	Tianjin
Li et al.[52]	1996–2012	STIRPAT	Tianjin
Tan et al.[53]	2000–2012	LMDI	Chongqing
Wang et al.[54]	2005–2010	LMDI	Suzhou
Chen et al.[55]	2000–2013	Decoupling Index	Macao
Liu et al.[56]	1995–2009	LMDI	Beijing, Shanghai, Tianjin, and Chongqing
Feng et al.[57]	2007	MRIO	Beijing, Shanghai, Tianjin, and Chongqing
Meng et al.[58]	2012	IO-LMDI	Beijing, Shanghai, Tianjin, and Chongqing

The second strand of research focuses on the city-level driving factors and influencing mechanisms. Decomposition analysis (e. g. Index Decomposition Analysis (IDA) and Structural Decomposition Analysis (SDA)) and regression method (e. g. Stochastic impacts by regression on population, affluence, and technology (STIRPAT) model) are the most commonly applied methods for the scientific evaluation and quantitative analysis of factors influencing city-level carbon emissions, especially the Logarithmic Mean Divisia Index (LMDI) method based on the IDA framework. As shown in Table 1, the SDA method based on the IO technique applied in the city-level driving factors and influencing mechanisms research in China were mainly focused on the four municipalities (i. e. Beijing, Shanghai, Tianjin and Chongqing), because of the available IO data base compiled by the Municipality Bureau of Statistics. In other words, the IO data sources were lacking in other provincial capital cities and medium-sized cities in China. Consequently, the IDA methods were used more frequently than the SDA methods based on IO technique [59–62], owing to its applicability in which each model can be applied to any available data at any aggregation level in a period-wise or time-series manner [63–65], where the preferred one in the LMDI [66–73]. By means of LMDI method, Wang et al. [46] investigated the main driver for industrial carbon emissions in Beijing-Tianjin-Hebei economic band from 1996 to 2010, and the results showed that economic output and energy intensity were both the most important influencing factors. Wang et al. [42] decomposed the influencing factors of residential carbon emissions in Beijing and found that the rising per capita GDP and the declining energy intensity contributed the most to emissions changes during 2000 to 2010. Similar results, conducted by Wang et al. [54], can also be found in Suzhou city. Zhao et al. [49] decomposed the main drivers for industrial carbon emissions in Shanghai and found that industrial output, energy intensity, energy and industrial structure were major determinants for emissions changes. Shao et al. [50] adopted an extended LMDI method and investigated the techno-economic drivers for industrial carbon emissions in Shanghai, and the results showed that industrial output scale was mainly responsible for emission increments while industrial structure adjustment, R&D intensity, and energy intensity were most significant factors in mitigating emissions. Kang et al. [51] performed a multi-sectoral decomposition analysis of city-level greenhouse gas emissions in Tianjin from 2001 to 2009, including the agricultural, industrial, transportation, commercial and other sectors, and the results showed that economic growth was the most important driver for emissions increments while energy efficiency was primarily responsible for emissions reductions. Hopefully, previous studies about driving factors and influencing mechanisms of city-level carbon emissions performed on China's megacities such as Beijing, Shanghai etc., have made great contributions to the realization of regional low-carbon road map. But, the previous LMDI studies mainly focused on the economic growth effect, the energy intensity effect, the population size effect, and the technology progress effect influencing the change of carbon emissions. It fails to distinguish the effects of economic structure, population structure, and energy structure on the change of carbon emissions. In fact, the strongest factors that offset China’s energy consumption and carbon emissions have shifted from efficiency gains to structural changes [74, 75]. Structural indicators combined with other traditional and emerging factors should also be deeply considered and investigated in China’s regional energy and carbon researches, which may perform different influencing mechanism in different regions during different development stages.

**Fig 1:**
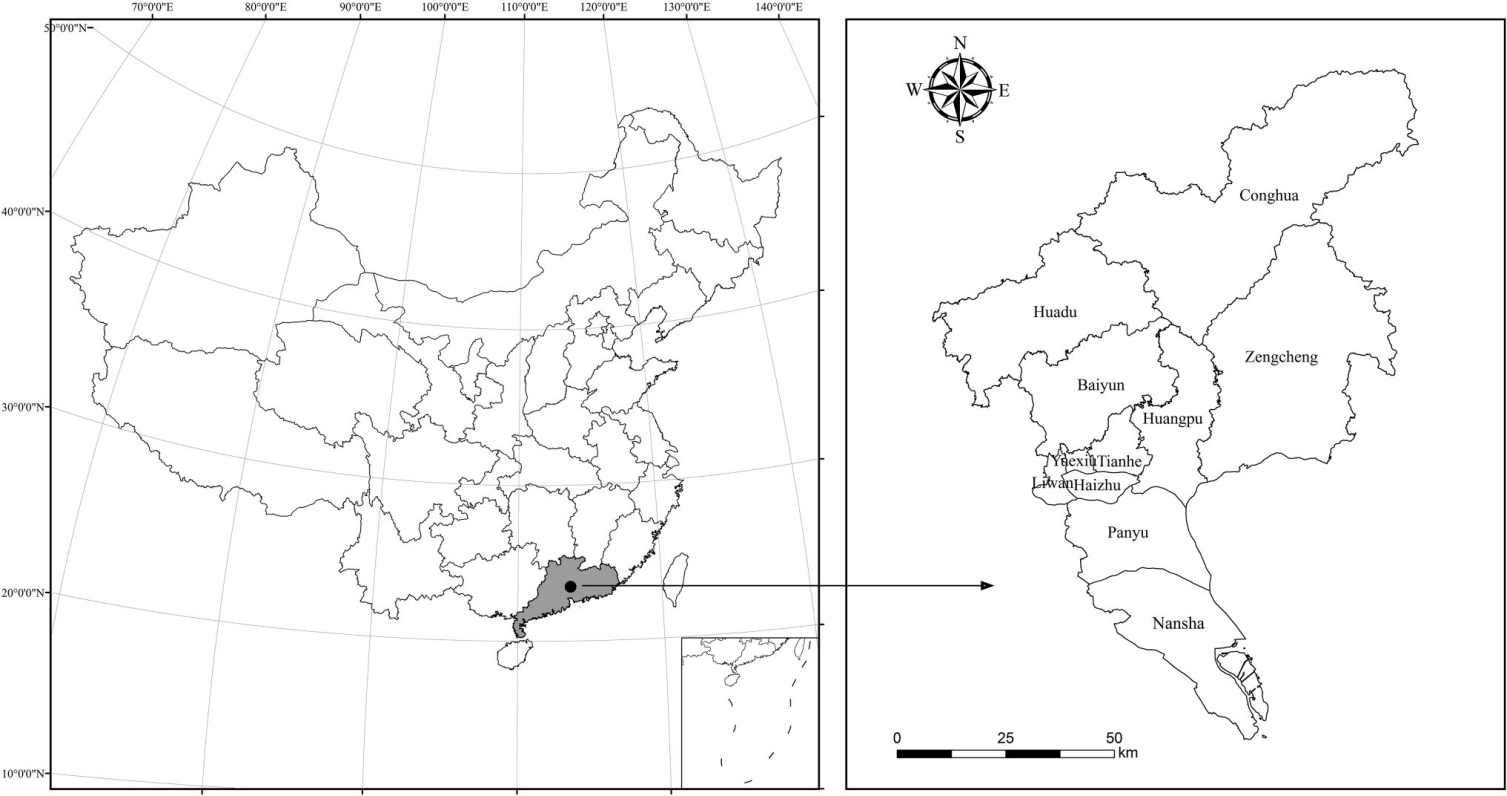
The location of Guangzhou city in China.

Compared with four municipalities, there were relatively few studies conducting on Guangzhou city, one of China’s first-tier cities, with the considerable GDP scale and total energy consumption amount (Table 2). Meanwhile, Guangzhou has the highest level of per capita GDP and per capita energy consumption than the four municipalities. Guangzhou is the provincial capital of Guangdong province, the most developed coastal region in the southeast of China (Fig 1). In addition, Guangzhou city is an important manufacturing base in China as well as a pilot zone for low-carbon city announced by China’s National Development and Reform Commission (NDRC) in 2012. Therefore, Guangzhou city may serve as a demonstration of how to realize the targets of low-carbon city development, thereby highlighting the importance and representativeness of studying its features and drivers for carbon emissions in detail.

**Table 2:** Socio-economic information and energy consumption of Guangzhou and other four municipalities in 2012.

	Beijing city	Shanghai city	Tianjin city	Chongqing city	Guangzhou city
Location	Municipality North	Municipality East	Municipality North	Municipality Southwest	Provincial capital Southeast
**Area (Sq.km)**	16410.54	6340.50	11916.85	82402.00	7434.40
**Population (Million)**	21.15	24.15	14.72	29.70	8.32
**GDP (Billion, Yuan)**	1950.06	2160.21	1437.02	1265.67	1542.01
**Energy consumption (Million tce)**	67.24	113.46	78.82	80.49	70.83
**Per capita GDP (Yuan/person)**	92201.42	89449.69	97623.64	42615.15	185337.74
**Per capita energy consumption (tce/person)**	3.18	4.70	5.35	2.71	8.51
**Energy consumption intensity (tce/10000 Yuan GDP)**	0.34	0.53	0.55	0.64	0.46
**Electricity consumption (Billion kilowatt-hour)**	90.87	141.06	79.45	81.33	71.07

The innovation and contribution of this study compared with other references mainly lies in the following two aspects. Firstly, city-level total carbon accounting approach based on the apparent energy consumption data from the comprehensive energy balance table was established. Secondly, driving factors including structural indicators, e. g. economic structure, energy structure, and population structure, were investigated systematically by adopting the newly extended LMDI method based on the Kaya identity and the IDA theory. It was aimed to provide theoretical references for making more targeted policies on energy saving and emission reduction in China’s mega cities.

This study is organized as follows: after the introduction, section 2 presents the methodology of city-level carbon accounting in Guangzhou and the method to decompose the changes in carbon emissions during the whole research period. Section 3 addresses the case analysis and the main results of Guangzhou city. Finally, we conclude our study and provide some policy implications for low-carbon city development.

## Methodology and data collection

### City-level total carbon accounting

As mentioned in Table 1, urban emissions inventory is the key for city-level total carbon accounting. However, there are accounting discrepancies between the different types of methods [21]. Based on China’s energy statistic and the reference approach of IPCC Guideline for National Greenhouse gas Emission Inventories, there are mainly three approach to account, i. e. primary energy consumption (e. g. coal, oil, natural gas, and renewables) [63], final energy consumption (e. g. coal, oil, natural gas, renewables, and electricity/heating) [61, 62], and total energy consumption from the “Energy Balance Table” including the aggregate summary of energy production, energy transformation and final consumption [76, 77]. Compared with the primary energy consumption accounting and the final energy consumption accounting, apparent energy consumption data [78, 79] from the comprehensive energy balance table was recommended for this study. As shown in the Table 3, the comprehensive energy balance table is mainly based on three modules, namely, 1) total energy available for local consumption as a result of interprovincial and international energy trades, 2) input-output of energy conversion transformation for thermal power, heating, coking, petroleum refining, and gas production, 3) final consumption due to primary industry, secondary industry, tertiary industry, and residential consumption. Primary industry, namely, agriculture, mainly includes farming, forestry, animal husbandry, and fishery. Secondary industry includes manufacturing industries and construction industry. Tertiary industry includes transportation, storage, post and telecommunication services, wholesale, retail trade and catering services, and other service sectors.

**Table 3:** Comprehensive energy balance table in Guangzhou in 2013 (Unit: Million tons of standard coal equivalent).

Item	Coal	Coke	Crude Oil	Gasoline	Diesel Oil	Fuel Oil	LPG	Electricity
**Total Energy Available for Local Consumption**	19.8885	0.3102	16.7568	4.2526	2.7134	4.692	3.1889	13.2952
Allocation from Other Provinces (cities)	19.7639	0.2456	0	13.3683	20.4704	32.7119	3.8337	13.118
Allocation to Other Provinces (-)	-0.344	0	0	-9.4359	-18.2254	-28.9829	-0.8366	0
Imports	0.3978	0	16.7265	0	0.0688	0.7129	0.1872	0
Exports (-)	0	0	0	0	-0.2087	-0.4469	0	0
**Input-Output of Energy Conversion Transformation**	-10.7529	0	-16.7379	3.1422	5.9872	0.0514	0.8788	10.7855
Thermal Power	-9.8499	0	0	0	-0.0025	-0.002	0	10.7855
Heating	-0.903	0	0	0	-0.0012	0	0	0
Coking	0	0	0	0	0	0	0	0
Petroleum Refining	0	0	-16.7379	3.1422	5.9909	0.0534	0.8788	0
Gas Production	0	0	0	0	0	0	0	0
**Losses**	0	0	0.0189	0	0	0	0	1.1941
**Final Consumption**	9.1356	0.3102	0	7.3948	8.7006	4.7434	4.0677	22.8866
Primary Industry	0.1099	0	0	0.1794	0.4854	0	0.1166	0.187
Secondary Industry	8.504	0.3102	0	0.5575	1.7583	2.0392	0.5427	11.4665
Tertiary Industry	0.5191	0	0	4.9262	6.4484	2.7042	2.3056	6.4423
Residential Consumption	0.0026	0	0	1.7317	0.0085	0	1.1028	4.7908
Urban Areas	0	0	0	1.5751	0	0	0.931	3.0671
Rural Areas	0.0026	0	0	0.1566	0.0085	0	0.1718	1.7237

Then, we calculated energy-related carbon emissions by energy types according to the IPCC Guidelines for National Greenhouse Gas Inventories based on the apparent energy consumption data from the comprehensive energy balance table, as follows the equation:

$$C_{t}=\sum_{i}E_{t}^{i}\times NCV_{i}\times CC_{t}^{i}\times O_{i}$$ (1)

Greenhouse gas (GHG) in our case study is referred in particular to carbon emissions-that directly affect climate change, which didn’t include other GHGs (i. e. CH_4_, N_2_O, etc.). Where the subscript *i* is the various fuels in this case study, *t* means the time in years. *C*_*t*_ represents total carbon emissions in year *t* (in million tons, Mt). $E_{t}^{i}$ represents the total energy consumption of fuel type *i* in year *t* (in million tons, Mt), and *NCV*_*i*_ is the net caloric value of fuel *i*. $CC_{t}^{i}$ is the carbon content of the fuel type *i*, and *O*_*i*_ is the oxidation rate of fuel *i*. The conversion factors, net caloric value, oxygenation efficiency and carbon content of the various fuels are listed in Table 4.

**Table 4:** Carbon emission factors of different energy sources.

Energy Sources	Conversion factors ^a^	NCV (PJ/10^4^ t, 10^8^m^3^) ^b^	Carbon Content (t C/TJ) ^b^	Oxygenation efficiency ^b^
**Raw coal**	0.714 t ce/t	0.20908	26.32	0.90
**Cleaned coal**	0.900 t ce/t	0.26344	26.32	0.90
**Other washed coal**	0.286 t ce/t	0.15000	26.32	0.90
**Coke**	0.971 t ce/t	0.28435	31.38	0.89
**Crude oil**	1.429 t ce/t	0.43000	20.08	0.96
**Gasoline**	1.471 t ce/t	0.44000	18.90	0.96
**Kerosene**	1.471 t ce/t	0.44000	19.60	0.96
**Diesel oil**	1.457 t ce/t	0.42652	20.20	0.96
**Fuel oil**	1.429 t ce/t	0.43000	21.10	0.96
**Nature gas**	1.330 t ce/10^3^ m^3^	3.89310	15.32	0.98
**LPG**	1.714 t ce/t	0.47000	20.00	0.97
**Refinery gas**	1.571 t ce/t	0.43000	20.20	0.97

^a^ Data resources:[63];

^b^ Data resources:[12]

### Data sources

Data sources, consisting of the gross domestic product including agriculture, production, construction, and service, the population including rural population and urban population, the total energy consumption based on industrial and residential perspective, covering the period from 2003 to 2013, were all available. Economic, population and energy data were collected from the Guangdong Province Statistical Yearbook (2003–2014) and Guangzhou City Statistical Yearbook (2003–2014). Economic data was measured by GDP in Chinese Yuan in time series. Energy data includes physical quantity of total energy consumption by fuel types measured by tons of standard coal equivalent (tce), which were compiled by the Guangdong Province Statistical Bureau and Guangzhou City Statistical Bureau.

### Data analysis

As mentioned and reviewed in Table 1, the LMDI methods based on the IDA theory, were used frequently to quantitatively analyze the drivers of carbon emissions at the city-level. The LMDI technique based on the IDA theory and an extended Kaya identity was conducted to uncover the main driving forces for energy-related carbon emissions in Guangzhou city.

The Kaya identity [18, 64, 80, 81] expresses carbon emissions as a product of four underlying driving factors:

$$C=P\times (\frac{G}{P})\times (\frac{E}{G})\times (\frac{C}{E})$$ (2)

Where *P* represents the population, *G* represents the GDP, *E* represents the total energy consumption; $\frac{G}{P}$ represents the per-capita GDP, $\frac{E}{G}$ represents the energy consumption intensity, and $\frac{C}{E}$ represents the carbon intensity of energy. Then, the Kaya identity was extended as follow:

$$\begin{array}{ll} C & =\sum_{k=1}^{n}C_{1}+\sum_{k=1}^{n}C_{2}+\sum_{k=1}^{n}C_{3}+\sum_{k=1}^{n}C_{4}+\sum_{k=1}^{n}C_{5}+\sum_{k=1}^{n}C_{6}\\ & =\sum_{k=1}^{n}\frac{C_{1}}{E_{1}}\times \frac{E_{1}}{GDP_{1}}\times \frac{GDP_{1}}{GDP}\times \frac{GDP}{P}\times P+\sum_{k=1}^{n}\frac{C_{2}}{E_{2}}\times \frac{E_{2}}{GDP_{2}}\times \frac{GDP_{2}}{GDP}\times \frac{GDP}{P}\times P\\ & +\sum_{k=1}^{n}\frac{C_{3}}{E_{3}}\times \frac{E_{3}}{GDP_{3}}\times \frac{GDP_{3}}{GDP}\times \frac{GDP}{P}\times P+\sum_{k=1}^{n}\frac{C_{4}}{E_{4}}\times \frac{E_{4}}{GDP_{4}}\times \frac{GDP_{4}}{GDP}\times \frac{GDP}{P}\times P\\ & +\sum_{k=1}^{n}\frac{C_{5}}{E_{5}}\times \frac{E_{5}}{TI_{urban}}\times AI_{urban}\times \frac{P_{urban}}{P}\times P+\sum_{k=1}^{n}\frac{C_{6}}{E_{6}}\times \frac{E_{6}}{TI_{rural}}\times AI_{rural}\times \frac{P_{rural}}{P}\times P \end{array}$$ (3)

Where $\sum_{k=1}^{n}C_{i}$ (*i* = 1, 2, 3, 4, 5, 6) represents carbon emissions from agriculture (*i* = 1), production (*i* = 2), construction (*i* = 3), service (*i* = 4), urban resident (*i* = 5), and rural resident (*i* = 6). *k* (*k* = 1, 2, 3, …*n*) represents the various energy types in this case study, mainly including coal, coke, crude oil, gasoline, diesel oil, kerosene, fuel oil, LPG, natural gas, and electricity. *E*_*i*_ (*i* = 1, 2, 3, 4, 5, 6) represents total energy consumption by different sectors. *GDP*_*i*_ (*i* = 1, 2, 3, 4) represents gross domestic product by different industries. *P*_*urban*_ and *P*_*rural*_ represent the total population of urban and rural residents, respectively. *TI*_*urban*_ and *TI*_*rural*_ represent the total income of urban and rural residents, respectively. *AI*_*urban*_ and *AI*_*rural*_ represent the average income of urban and rural residents, respectively.

Then, Eq (3) can be rewritten as follow:

$$\begin{array}{ll} C & =f_{1}\times e_{1}\times s_{1}\times g\times p+f_{2}\times e_{2}\times s_{2}\times g\times p\\ & +f_{3}\times e_{3}\times s_{3}\times g\times p+f_{4}\times e_{4}\times s_{4}\times g\times p\\ & \begin{array}{l} +f_{5}\times e_{5}\times AI_{urban}\times UR\times p+f_{6}\times e_{6}\times AI_{rural}\times (1-UR)\times p \end{array} \end{array}$$ (4)

Where *p* = *P*, represents the total population size, *UR* represents the level of urbanization.

$g=\frac{GDP}{P}$, represents the per capita GDP.

$e_{i}=\frac{E_{i}}{GDP_{i}}$ (*i* = 1, 2, 3, 4, 5, 6), represents the energy consumption intensity of agriculture, production, construction, service, urban resident, and rural resident, respectively.

$f_{i}=\frac{C_{i}}{E_{i}}$ (*i* = 1, 2, 3, 4, 5, 6), represents the energy carbon intensity of agriculture, production, construction, service, urban resident, and rural resident, respectively.

$s_{i}=\frac{GDP_{i}}{GDP}$ (*i* = 1, 2, 3, 4), represents the economic structure of agriculture, production, construction, and service, respectively.

Then, the changes of regional carbon emissions between two years can be decomposed as:

$$\begin{array}{ll} \Delta C & =C^{T}-C^{0}\\ & =\Delta C_{p}+\Delta C_{g}+\Delta C_{e_{i}}+\Delta C_{f_{i}}+\Delta C_{s_{i}}+\Delta C_{AI_{urban}}+\Delta C_{AI_{rural}}+\Delta C_{UR}+\Delta C_{P_{rural}} \end{array}$$ (5)

Following the preferred LMDI method proposed by Ang et al. [66, 82, 83], the various effects of each factor can be quantificationally calculated as:

$$\begin{array}{ll} \Delta C & =\sum_{i=1}^{6}\left(\frac{C_{i}^{t}-C_{i}^{0}}{\text{ln}C_{i}^{t}-\text{ln}C_{i}^{0}}\text{ln}(\frac{f_{i}^{t}}{f_{i}^{0}})\right)+\sum_{i=1}^{4}\left(\frac{C_{i}^{t}-C_{i}^{0}}{\text{ln}C_{i}^{t}-\text{ln}C_{i}^{0}}\text{ln}(\frac{s_{i}^{t}}{s_{i}^{0}})\right)\\ & +\sum_{i=1}^{6}\left(\frac{C_{i}^{t}-C_{i}^{0}}{\text{ln}C_{i}^{t}-\text{ln}C_{i}^{0}}\text{ln}(\frac{p_{}^{t}}{p_{}^{0}})\right)+\sum_{i=1}^{4}\left(\frac{C_{i}^{t}-C_{i}^{0}}{\text{ln}C_{i}^{t}-\text{ln}C_{i}^{0}}\text{ln}(\frac{g_{i}^{t}}{g_{i}^{0}})\right)\\ & +\frac{C_{5}^{t}-C_{5}^{0}}{\text{ln}C_{5}^{t}-\text{ln}C_{5}^{0}}\text{ln}(\frac{AI_{urban}^{t}}{AI_{urban}^{0}})+\frac{C_{6}^{t}-C_{6}^{0}}{\text{ln}C_{6}^{t}-\text{ln}C_{6}^{0}}\text{ln}(\frac{AI_{rural}^{t}}{AI_{rural}^{0}})\\ & +\frac{C_{5}^{t}-C_{5}^{0}}{\text{ln}C_{5}^{t}-\text{ln}C_{5}^{0}}\text{ln}(\frac{UR_{}^{t}}{UR_{}^{0}})+\frac{C_{6}^{t}-C_{6}^{0}}{\text{ln}C_{6}^{t}-\text{ln}C_{6}^{0}}\text{ln}(\frac{P_{rural}^{t}}{P_{rural}^{0}})\\ & +\frac{C_{1}^{t}-C_{1}^{0}}{\text{ln}C_{1}^{t}-\text{ln}C_{1}^{0}}\text{ln}(\frac{e_{1}^{t}}{e_{1}^{0}})+\frac{C_{2}^{t}-C_{2}^{0}}{\text{ln}C_{2}^{t}-\text{ln}C_{2}^{0}}\text{ln}(\frac{e_{2}^{t}}{e_{2}^{0}})+\frac{C_{3}^{t}-C_{3}^{0}}{\text{ln}C_{3}^{t}-\text{ln}C_{3}^{0}}\text{ln}(\frac{e_{3}^{t}}{e_{3}^{0}})\\ & +\frac{C_{4}^{t}-C_{4}^{0}}{\text{ln}C_{4}^{t}-\text{ln}C_{4}^{0}}\text{ln}(\frac{e_{4}^{t}}{e_{4}^{0}})+\frac{C_{5}^{t}-C_{5}^{0}}{\text{ln}C_{5}^{t}-\text{ln}C_{5}^{0}}\text{ln}(\frac{e_{5}^{t}}{e_{5}^{0}})+\frac{C_{6}^{t}-C_{6}^{0}}{\text{ln}C_{6}^{t}-\text{ln}C_{6}^{0}}\text{ln}(\frac{e_{6}^{t}}{e_{6}^{0}}) \end{array}$$ (6)

In the newly established LMDI method, the population effect (Δ*C*_*p–effect*_), the affluence effect (Δ*C*_*g–effect*_), the energy intensity effect (Δ*C*_*e–effect*_), the economic structure effect (Δ*C*_*s–effect*_), and the carbon intensity effect (Δ*C*_*f–effect*_). In addition, social and economic factors such as urbanization (Δ*C*_*UR–effect*_), family income in urban and rural households (Δ*C*_*AI–effect*_) were analyzed to further explain the changes of carbon emissions at the city level.

## Empirical analysis in Guangzhou

### Total energy consumption and economic development in Guangzhou

**Fig 2:**
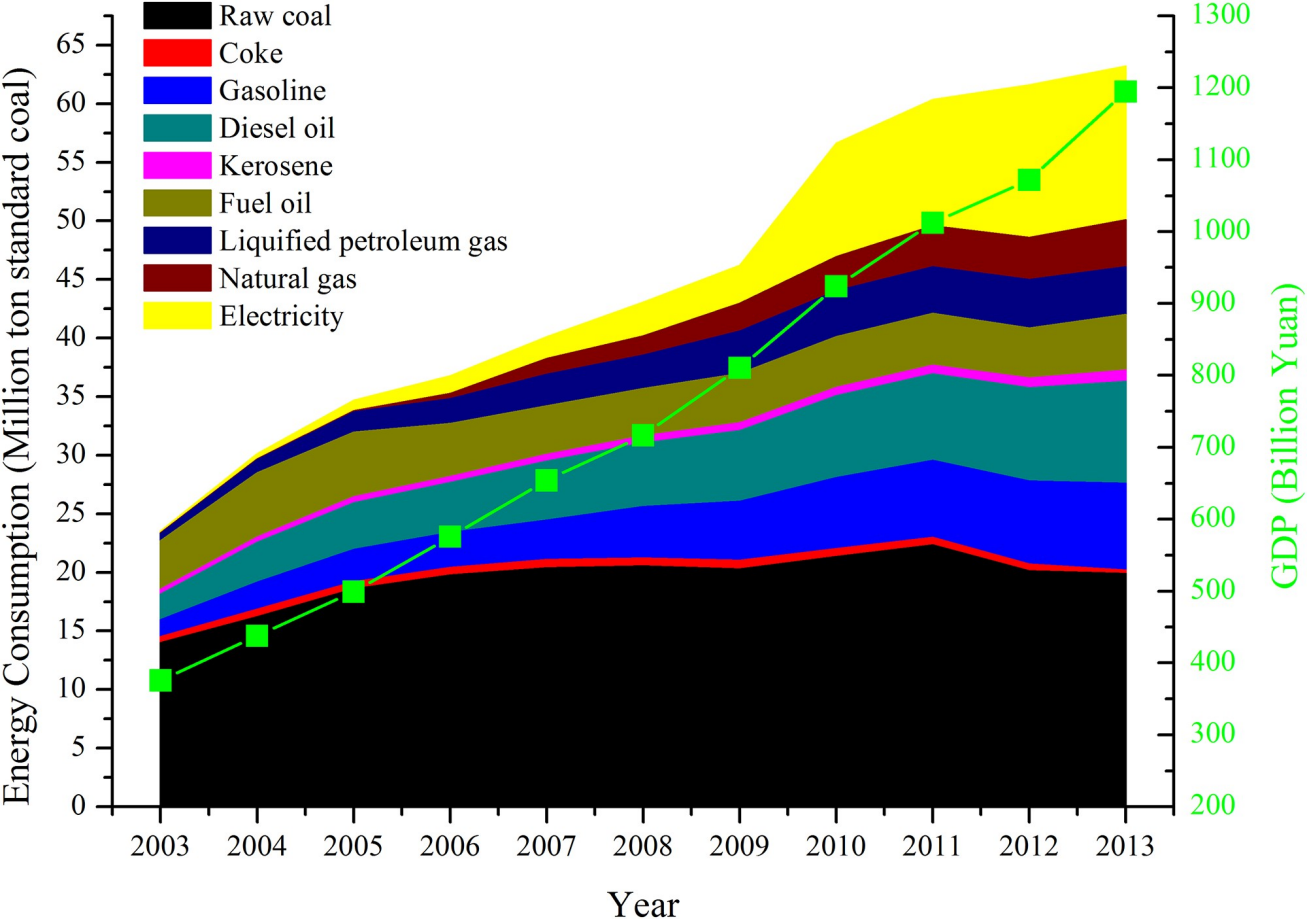
Total energy consumption and GDP in Guangzhou during 2003 to 2013.

Economic development in Guangzhou was described with GDP, which was converted into the 2003 constant prices (Fig 2). GDP of Guangzhou increased from 375.86 billion Yuan in 2003 to 1194.74 billion Yuan in 2013 at an average annual growth rate of 11.09%. Meanwhile, per capita GDP in Guangzhou grew from 38631.93 Yuan in 2003 to 92423.82 Yuan in 2013 (at the fixed price in 2003), which was 2.39 times as higher as in 2003 after ten years of rapid economic development.

Along with the rapid economic development, total energy consumption increased rapidly from 23.529 million tons of coal equivalent (tce) in 2003 to 63.198 million tce in 2013, showing an average annual growth rate of 9.39% over a period of 11 years. However, there were various growth trends existing in the main 9 types of energy sources over the research period. The leading energy in Guangzhou over the whole research period is still coal (raw coal plus coke), which increased from 14.494 million tce in 2003 to 22.993 million tce in 2011, then decreased to 20.198 million tce in 2013. Oil products such as gasoline and diesel oil also play an important role in Guangzhou’s energy consumption system. Gasoline and diesel oil had an overall growing trend, which grew from 3.636 million tce in 2003 to 16.099 million tce in 2013. In addition, the most important features of energy consumption structure in Guangzhou, is the electricity consumption. The electricity consumption as shown in Fig 2, is not the thermal power or renewable energy power generated in Guangzhou, which represented the imported electricity from the neighbors of Guangdong province—Yunnan province, Guizhou province, and Guangxi province etc. The imported electricity was calculated from the comprehensive energy balance table in Guangzhou. The imported electricity increased rapidly from 0.173 million tce in 2003 to 13.118 million tce in 2013, which played an important role in the energy consumption system in Guangzhou. As shown in Fig 2, there was a sharply increase in the imported electricity in Guangzhou after 2009. The rapid increase in the imported electricity resulted in the sluggish and even decrease in fossil energy consumption in Guangzhou to a great extent in recent years. In fact, the increased imported electricity consumed in Guangzhou was benefited by the “*West-East electricity transmission project*” [84] conducting by China’s central government after 2000. The similar condition was familiar in other mega cities in China such as Beijing, Shanghai, and Tianjin etc [14].

### Carbon emissions evolution and carbon emissions structure in Guangzhou

**Fig 3:**
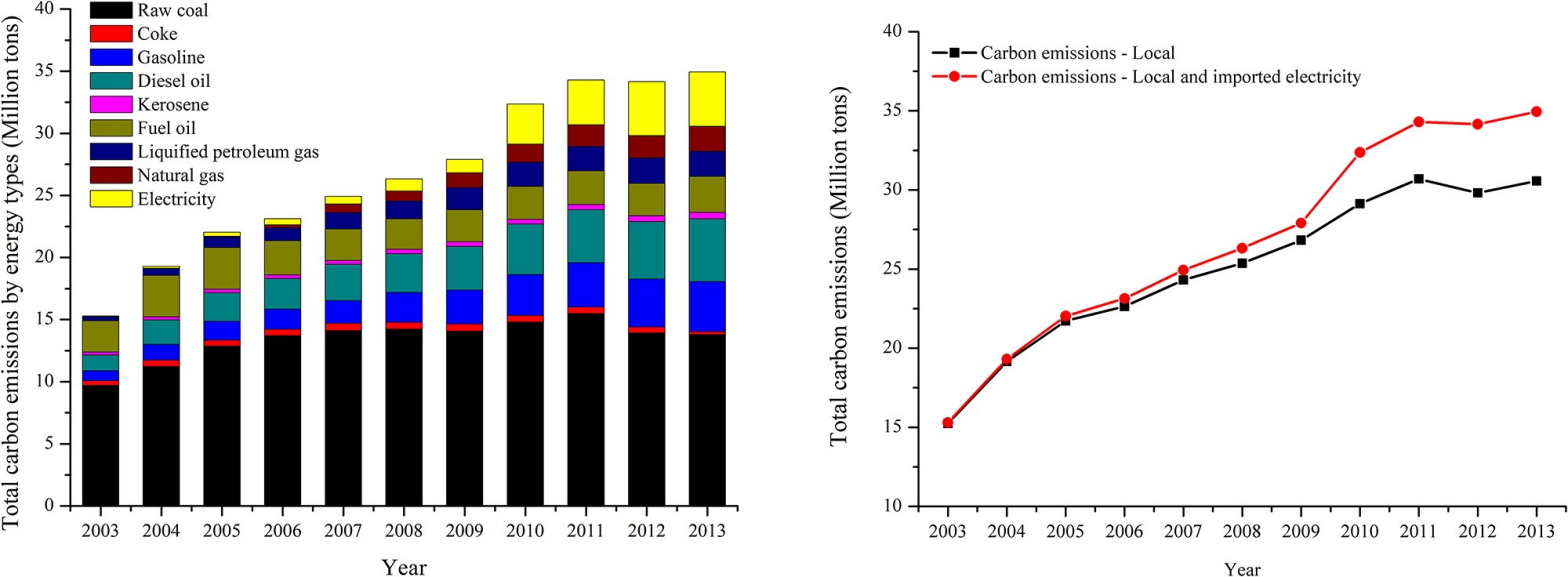
Carbon emissions structure (left) and carbon emissions evolution (right) in Guangzhou during 2003 to 2013.

According to the urban emissions inventory in Guangzhou and Eq (1), we calculated the energy-related carbon emissions from 2003 to 2013. Total energy-related carbon emissions mainly include two parts, namely, carbon emissions generated in the boundary from fossil fuel combustion and out of the boundary embodied in the imported electricity (Fig 3). Carbon emissions embodied in the imported electricity need to be calculated by considering the total amount of imported electricity and the corresponding emissions factor of power generation [56]. As for China’s power supply system, electricity is supplied by the main six regional grids consisting of *North China Grid*, *Northeast China Grid*, *East China Grid*, *Central China Grid*, *Northwest China Grid*, and *South China Grid*. Guangzhou city is supplied by the *South China Grid* which covered Guangdong, Guangxi, Yunnan and Guizhou provinces. According to the “*West-East electricity transmission project*”, the southern route of this project refers to transmitting Guizhou’s thermal power and Yunnan, Guizhou, Guangxi’s hydropower to Guangdong, Hong Kong, and Macau [84]. At present, Guangdong’s imported electricity was mainly from Guizhou and Yunnan provinces. More than 60 percent of the cross-province electricity trades were thermal power, and less than 40 percent were hydropower, based on our estimation according to the relevant government documents (e. g. The *12*^*th*^ *five-year plan for energy development in Guangdong province* [85], and the *12*^*th*^ *five-year plan for power industry development in Guizhou province* [86]). The corresponding emissions factor of power generation in *South China Grid* can be found in the *baseline emission factors for China's regional grid* announced by the National Development and Reform Commission.

As shown in Fig 3, there were obviously two different carbon emissions evolution processes in Guangzhou, namely, the first stage (2003–2009) and the second stage (2010–2013). During the first stage, carbon emissions generated in the boundary from fossil fuel combustion increased rapidly from 15.243 million tons in 2003 to 26.822 million tons in 2009 with an annual growth rate of 8.41%. Raw coal is still the biggest contributor to the total carbon emissions, which was followed by diesel oil, gasoline, and fuel oil. But the share of raw coal contributing to the total emissions has decreased dramatically from 63.69% in 2003 to 52.45% in 2009. During the same period, carbon emissions embodied in the imported electricity increased from 0.057 million tons in 2003 to 1.080 million tons in 2009. And the share of imported electricity accounting for the total emissions has decreased from 0.37% in 2003 to 3.87% in 2009. Imported electricity has played an important but relatively minor effect on the total carbon emissions in Guangzhou during this period. During the second stage, carbon emissions generated in the boundary from fossil fuel combustion increased slowly from 29.130 million tons in 2010 to 30.565 million tons in 2013 with an annual growth rate of 1.21%, and even decreased from 30.693 million tons in 2011 to 29.811 million tons in 2012. Raw coal is still the biggest contributor to the total carbon emissions during this period. In such a case, Guangzhou perfectly completed the energy saving and emission reduction targets (i. e. the energy consumption and carbon dioxide emissions per unit GDP decreased by 16% and 17% during the 12^th^ five-year plan period, respectively.), which were issued by the central government and performed by the local government. But in fact, carbon emissions mitigation in the boundary mainly benefited from contributions of the imported electricity from the neighbors Guizhou and Yunnan provinces via the cross-province electricity trades. During the same period, carbon emissions embodied in the imported electricity dramatically increased 3.236 million tons in 2010 to 4.379 million tons in 2013. Rapidly increasing carbon emissions embodied in imported electricity indicated that there were large amounts of fossil energy were burned for power. Final products-thermal power was consumed in Guangzhou, while carbon emissions were discharged in its neighbor producers. If these carbon emissions embodied in the imported electricity were allocated to the final consumer, Guangzhou would have paid more efforts and implemented more strict mitigation measures to achieve its energy saving and emission reduction targets.

### Historical decomposition analysis of carbon emissions in Guangzhou

Aiming to have a better understanding of the complex various influencing factors for carbon emissions in Guangzhou city, we decomposed the total carbon emissions into two parts including the industrial sector and the residential sector.

#### Driving factors of carbon emissions in industrial sector in Guangzhou

**Fig 4:**
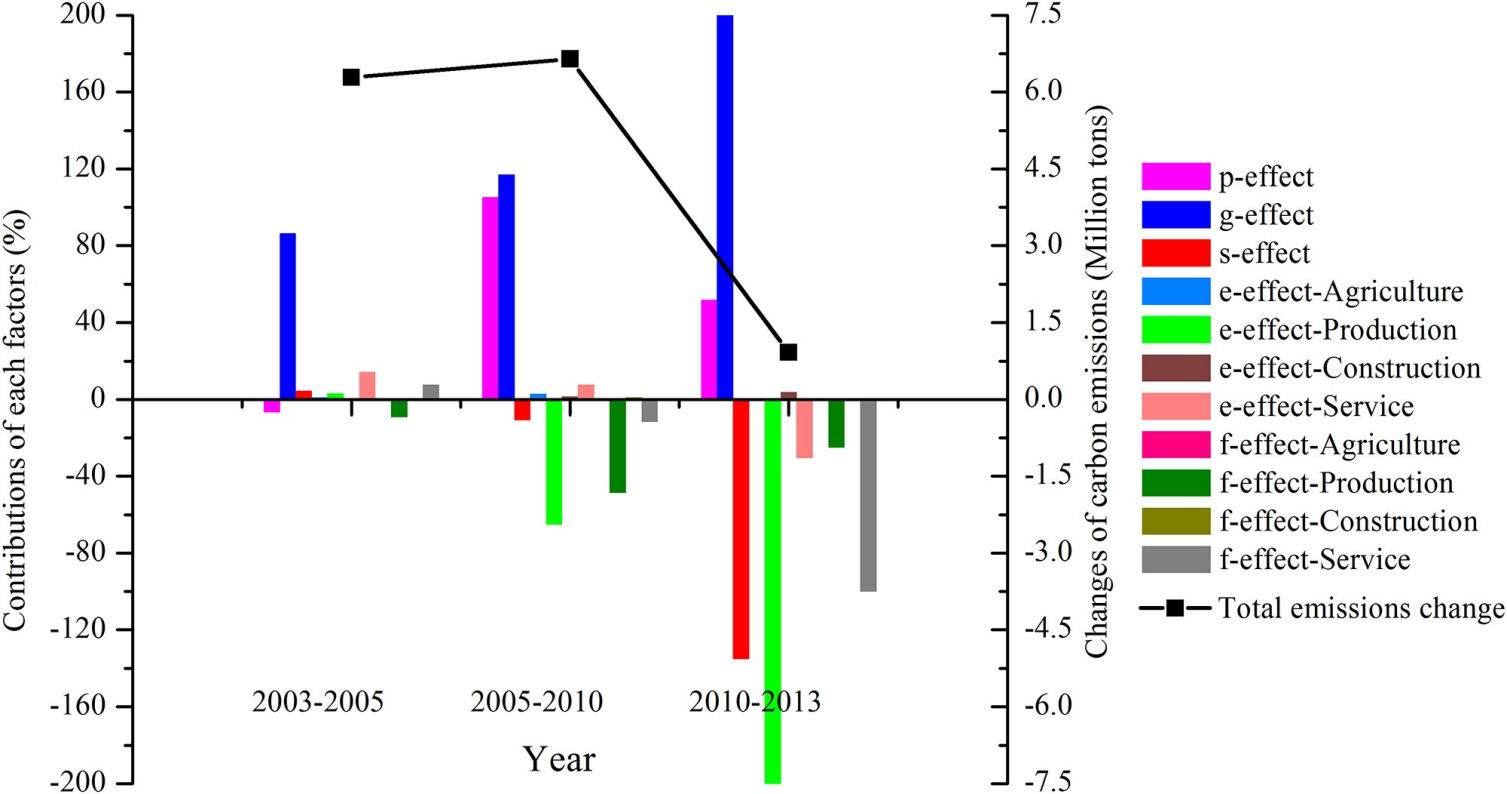
Contributions of each factors and total carbon emission change in industrial sector in Guangzhou in million tones over 2003–2013.

Driving factors of carbon emissions in industrial sector were decomposed yearly first (Table 5). In order to have a better understanding of influencing factors in long time series, we divided the carbon emissions process into 3 periods (Fig 4), according to the regional five-year plans for socio-economic development, combined with a certain historical background.

**Table 5:** Decomposition of carbon emission change in industrial sector in Guangzhou in million tones (2003–2013).

Period	*p-effect*	*g-effect*	*s-effect*	*e-effect-1*	*e-effect-2*	*e-effect-3*	*e-effect-4*	*f-effect-1*	*f-effect-2*	*f-effect-3*	*f-effect-4*	*ΔCE-Industry*
**2003–2004**	-0.1168	2.6246	0.2621	0.0708	0.0398	0.0074	0.8847	-0.0328	-0.5332	-0.0078	0.6152	3.8139
**2004–2005**	-0.3372	2.9386	0.0079	-0.0148	0.1579	0.0133	-0.0481	-0.0181	-0.0036	-0.0071	-0.2141	2.4747
**2005–2006**	0.5714	2.4739	0.3141	0.0124	-2.0603	0.0001	-0.2971	0.0185	-0.0217	0.0064	-0.3059	0.7119
**2006–2007**	0.6563	2.1770	-0.0449	0.0944	-1.2670	0.0977	0.0515	0.0561	-0.8456	0.0528	0.2802	1.3085
**2007–2008**	2.4632	-0.3057	-0.2150	0.0236	-0.6157	0.0044	0.1300	-0.0091	0.0258	-0.0077	-0.3858	1.1081
**2008–2009**	1.5475	1.5131	-0.6874	-0.0022	-0.6684	-0.0104	-0.2905	-0.0098	-0.0725	0.0027	0.2059	1.5279
**2009–2010**	1.8188	1.6604	-0.1017	0.0719	0.4027	-0.0061	1.2054	-0.0457	-2.4174	-0.0151	-0.5815	1.9916
**2010–2011**	0.0925	2.4797	-0.2491	0.0161	-0.5724	-0.0117	-0.1638	-0.0174	-0.2054	-0.0118	-0.2374	1.1194
**2011–2012**	0.1935	1.4291	-0.7204	0.0007	-0.7123	0.0019	-0.0757	-0.0001	-0.7842	0.0033	-0.2259	-0.8902
**2012–2013**	0.1924	2.8672	-0.3020	-0.0158	-2.3444	0.0485	-0.0295	0.0191	0.7124	0.0067	-0.4635	0.6910
**2003–2013**	5.7907	17.7760	-1.5181	0.2815	-6.4647	0.1876	1.7101	-0.0402	-3.5047	0.0298	-0.3910	13.8569

During the 10^th^ five-year plan period (2003–2005), Guangzhou announced the overall objective in the 10^th^ five-year plan that it would *take the lead in realizing socialist modernization and building the modernization central city* over China. Guangzhou’s industrial carbon emissions increased by 6.29 million tons mainly because the rapid economic development. The affluence effect (***g-effect***) was the dominant positive effect in driving carbon emissions increase, bringing in a 5.43 million tons increase, accounting for 86.34% of the total industrial carbon emissions changes, which was followed by the energy intensity effect of service (***e-effect-Service***), the carbon intensity effect of service (***f-effect-Service***), and the economic structure effect (***s-effect***). In order to achieve the basic standards of modernization (i. e. per capita GDP is more than 5000 U.S. dollar, and the third industry added value accounted for more than 50% of GDP etc.), Guangzhou paid more efforts to foster the three pillar industries (i. e. electronic information manufacturing industry, automobile industry, and petrochemical industry) and accelerate the development of the third industry (i. e. transportation industry and logistics industry). The proportion of production industry increased from 34.96% in 2003 to 35.77% in 2005. The energy intensity of service increased from 0.026 tce/thousand Yuan in 2003 to 0.032 tce/thousand Yuan in 2005. The carbon intensity effect of production (***f-effect-Production***) played the most important role in curbing carbon emissions growth, resulting in a 5.77 million tons decrease, accounting for 9.18% of the total industrial carbon emissions changes in absolute value, which was followed by the population effect (***p-effect***).

During the 11^th^ five-year plan period (2005–2010), Guangzhou’s industrial carbon emissions increased by 6.64 million tons mainly because the rapid economic development and population growth. The affluence effect (***g-effect***) and population effect (***p-effect***) were the most two dominant positive driving factors on the carbon emissions increment, bringing in 7.78 million tons and 6.99 million tons increase, accounting for 117.00% and 105.28% of the total industrial carbon emissions changes, respectively. During 2005 to 2010, GDP in Guangzhou still increased rapidly from 515.42 billion Yuan in 2005 to 1074.83 billion Yuan in 2010 with an annual average growth rate of 15.83%. Per capita GDP also increased rapidly from 52582.27 Yuan/person in 2005 to 72690.13 Yuan/person in 2010. The total population stopped falling from 9.72 million in 2003, 9.66 million in 2004, to 9.49 million in 2005, which was because of the outbreak of the serious infectious disease named *Severe Acute Respiratory Syndrome (SARS)* occurred in Guangzhou in 2003. The population effect (***p-effect***) changed from negative to positive during 2005 to 2010. The total population increased from 9.75 million in 2006 to 12.71 million in 2010, which played the second important role in the carbon emissions growth. The energy intensity effect of production (***e-effect-Production***) and the carbon intensity effect of production (***f-effect-Production***) played the most two important roles in curbing carbon emissions growth, resulting in 4.32 million tons and 3.23 million tons decrease, accounting for 64.96% and 48.62% of the total industrial carbon emissions changes in absolute value, which was followed by the carbon intensity effect of service (***f-effect-Service***), and the economic structure effect (***s-effect***). The adjustment of industrial structure in Guangzhou was mainly focused on upgrading the traditional advantage industries (i. e. iron and steel, shipbuilding, machinery and equipment, electricity, paper, and textile etc.) and fostering the new and high technology industries (i. e. Software, new material, new energy, and digital industry etc.), announced in the *11*^*th*^ *five-year plan for socioeconomic development in Guangzhou*. The proportion of production industry decreased from 35.77% in 2005 to 33.91% in 2010. The energy intensity of production decreased from 0.117 tce/thousand Yuan in 2005 to 0.090 tce/thousand Yuan in 2010. The carbon intensity of production sharply decreased from 0.758 in 2005 to 0.625 in 2010. It indicates that the adjustment of industrial structure achieved effectively to curb carbon emissions during this period.

During the 12^th^ five-year plan period (2010–2013), the growth rate of Guangzhou’s industrial carbon emissions decreased obviously compared with the previous two periods. The total industrial carbon emissions only increased by 9.20 million tons during 2010 to 2013. The affluence effect (***g-effect***) was the most dominant driving factors on the carbon emissions increment, bringing in a 6.73 million tons increase, accounting for 732.04% of the total industrial carbon emissions changes, which was followed by the population effect (***p-effect***). During 2010 to 2013, GDP in Guangzhou still increased rapidly from 1074.83 billion Yuan in 2010 to 1549.72 billion Yuan in 2013. Per capita GDP also increased rapidly from 72690.13 Yuan/person in 2010 to 92423.82 Yuan/person in 2013. The energy intensity effect of production (***e-effect-Production***) and the economic structure effect (***s-effect***) played the most two important roles in curbing carbon emissions growth, resulting in 3.65 million tons and 1.24 million tons decrease, accounting for 396.81% and 135.08% of the total industrial carbon emissions changes in absolute value, which was followed by the carbon intensity effect of service (***f-effect-Service***), the energy intensity effect of service (***e-effect-Service***), and the carbon intensity effect of production (***f-effect-Production***). The proportion of production industry decreased steadily from 33.91% in 2010 to 30.71% in 2013. The energy intensity of production decreased from 0.090 tce/thousand Yuan in 2010 to 0.072 tce/thousand Yuan in 2013. The carbon intensity of service sharply decreased from 0.440 in 2010 to 0.401 in 2013. The energy intensity of service decreased from 0.036 tce/thousand Yuan in 2010 to 0.035 tce/thousand Yuan in 2013. The carbon intensity of production decreased from 0.625 in 2010 to 0.617 in 2013. It indicates that the adjustment of industrial structure achieved effectively to curb carbon emissions during this period. It indicates that the adjustment of industrial structure and energy consumption structure effectively achieved to curb carbon emissions during this stage.

#### Driving factors of carbon emissions in residential sector in Guangzhou

**Fig 5:**
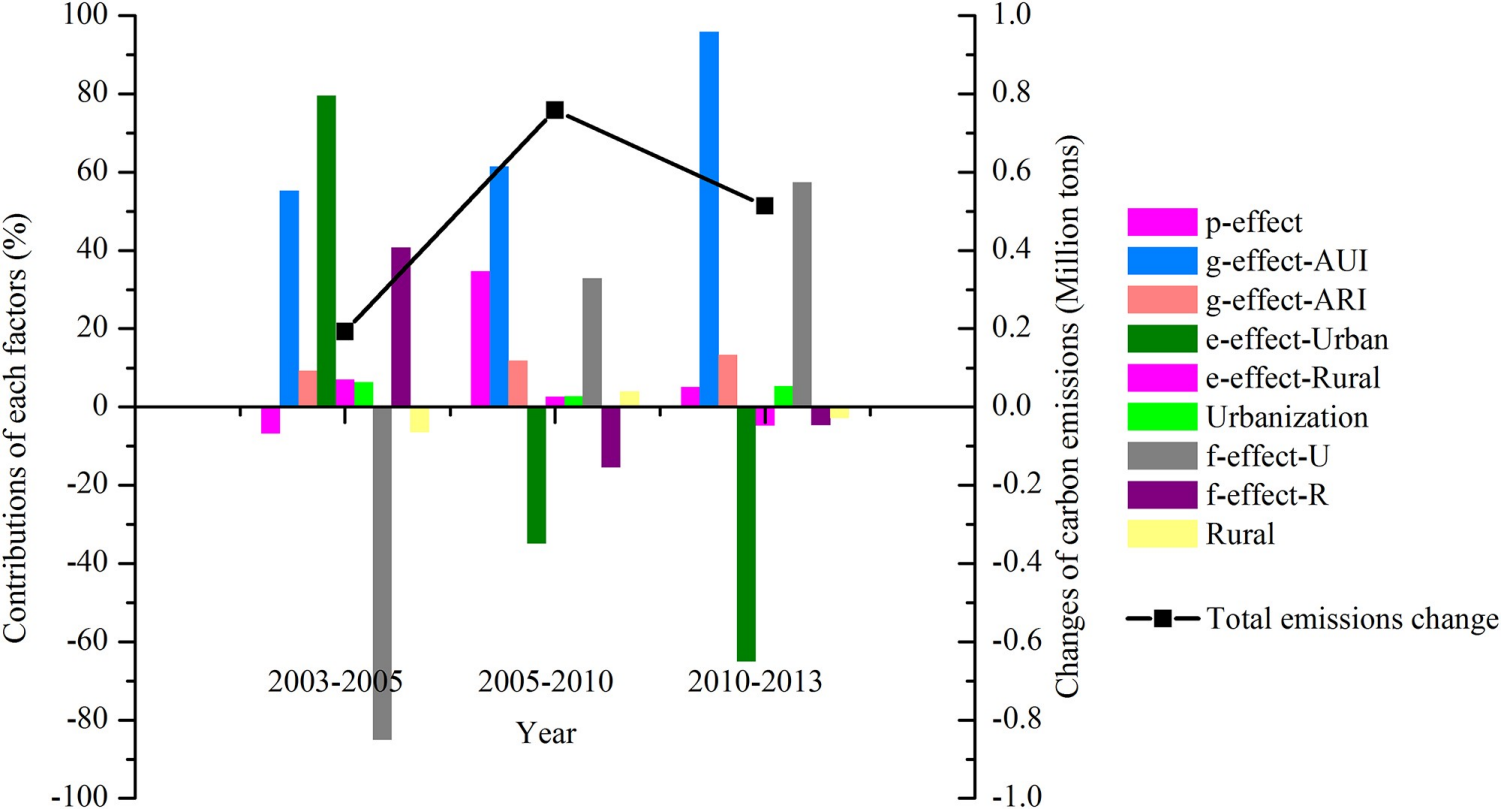
Contributions of each factors and total carbon emission change in residential sector in Guangzhou in million tones over 2003–2013.

Driving factors of carbon emissions in residential sector were decomposed yearly first (Table 6) and divided into 3 periods (Fig 5). Although the residential carbon emissions in Guangzhou accounted for a relatively small proportion of the total carbon emissions, it exhibited a rapid growth trend during the whole research period along with the improvement of urbanization level. The total residential carbon emissions increased from 0.55 million tons in 2003 accounting for 3.61% of the total carbon emissions to 2.01 million tons accounting for 6.59% in 2013. Furthermore, the residential carbon emission increments were mainly generated by the urban residents, which increased from 0.49 million tons in 2003 to 1.83 million tons in 2013.

**Table 6:** Decomposition of carbon emission change in residential sector in Guangzhou in million tones (2003–2013).

	*p-effect*	*g-effect-AUI*	*g-effect-ARI*	*e-effect-U*	*e-effect-R*	*f-effect-U*	*f-effect-R*	*Urbanization*	*Rural*	*ΔCE-Resident*
**2003–2004**	-0.0036	0.0597	0.0058	0.0462	0.0238	-0.0774	0.0393	0.0072	-0.0059	0.0952
**2004–2005**	-0.0095	0.0441	0.0153	0.1072	-0.0193	-0.0838	0.0457	0.0047	-0.0077	0.0969
**2005–2006**	0.0178	0.0546	0.0120	-0.0196	0.0267	0.1143	-0.0113	0.0014	0.0030	0.1989
**2006–2007**	0.0273	0.1150	0.0198	-0.0896	-0.0113	0.3204	-0.0185	0.0015	0.0041	0.3686
**2007–2008**	0.1159	0.1322	0.0181	-0.2356	0.0086	-0.0523	-0.0641	0.0008	0.0174	-0.0589
**2008–2009**	0.0654	0.0911	0.0169	-0.0115	-0.0089	-0.2229	-0.0083	0.0038	0.0075	-0.0668
**2009–2010**	0.0793	0.1216	0.0257	0.0656	0.0046	0.0211	-0.0172	0.0174	-0.0010	0.3171
**2010–2011**	0.0050	0.1779	0.0232	-0.0491	-0.0011	0.3018	-0.0176	0.0064	-0.0034	0.4432
**2011–2012**	0.0121	0.1761	0.0236	-0.1026	0.0014	-0.0997	-0.0120	0.0186	-0.0094	0.0080
**2012–2013**	0.0123	0.1794	0.0217	-0.2223	-0.0251	0.0871	0.0062	0.0053	-0.0019	0.0628
**2003–2013**	0.2887	1.0471	0.1209	-0.2252	0.0129	0.1631	-0.0048	0.0646	-0.0023	1.4649

During the 10^th^ five-year plan period (2003–2005), Guangzhou’s residential carbon emissions increased from 0.55 million tons in 2003 to 0.74 million tons in 2013. The energy intensity effect of urban (***e-effect-Urban***) and the affluence effect of urban (***g-effect-AUI***) were the most two dominant positive driving factors on the carbon emissions increment, bringing in 0.15 million tons and 0.11 million tons increase, accounting for 79.60% and 55.22% of the total residential carbon emissions changes, respectively. The carbon intensity effect of urban (***f-effect-Urban***) played the most important role in curbing carbon emissions growth, resulting in a 0.16 million tons decrease, accounting for 84.98% of the total residential carbon emissions changes in absolute value.

During the 11^th^ five-year plan period (2005–2010), Guangzhou’s residential carbon emissions increased by 0.75 million tons mainly because of the rapid growth of the average income of urban residents during the urbanization process. The affluence effect of urban (***g-effect-AUI***) was the most dominant positive driving factor on the carbon emissions increment, bringing in a 0.47 million tons increase, accounting for 61.48% of the total residential carbon emissions changes, which was followed by the population effect (***p-effect***) and the carbon intensity effect of urban (***f-effect-Urban***). The average income of urban residents in Guangzhou increased from 18287.24 Yuan/person in 2005 to 30658.49 Yuan/person in 2010, which was always two times of the average income of rural residents during the same period. The energy intensity effect of urban (***e-effect-Urban***) played the most important role in curbing carbon emissions growth, resulting in a 0.26 million tons decrease, accounting for 34.87% of the total residential carbon emissions changes in absolute value.

During the 12^th^ five-year plan period (2010–2013), Guangzhou’s residential carbon emissions increased by 0.51 million tons. The affluence effect of urban (***g-effect-AUI***) and the carbon intensity effect of urban (***f-effect-Urban***) were the most two dominant positive driving factors on the carbon emissions increment, bringing in 0.49 million tons and 0.29 million tons increase, accounting for 95.88% and 57.43% of the total residential carbon emissions changes, respectively.

The energy intensity effect of urban (***e-effect-Urban***) played the most important role in curbing carbon emissions growth, resulting in a 0.33 million tons decrease, accounting for 64.95% of the total residential carbon emissions changes in absolute value.

## Main conclusions and policy recommendations

### Main conclusions

Based on the apparent energy consumption data from the comprehensive energy balance table, a systematic and comprehensive city-level total carbon accounting approach was established and applied in one of the China’s mega city—Guangzhou. Total carbon emissions including the fossil fuel combustion in the city’s boundary and embodied in the imported electricity out of the boundary were still performing a rising tendency. Carbon emissions embodied in the imported electricity played a significant important role in emissions mitigation in Guangzhou, especially after 2009. Examining the effects of different boundaries on carbon accounting at city level is crucial for the emissions mitigation in urban China.

Then, the newly extended LMDI method based on the Kaya identity was adopted to examine the main drivers for the carbon emissions increments both at the industrial sector and the residential sector in Guangzhou city. Quantitative analyses and time-series analysis were performed on the influencing mechanism for various influencing factors both at the industrial sector and the residential sector for the three different development periods, namely, the 10^th^ five-year plan period (2003–2005), the 11^th^ five-year plan period (2005–2010), and the 12^th^ five-year plan period (2010–2013). The influences and impacts of various driving factors on industrial and residential carbon emissions are different in the three different development periods. Overall, the affluence effect (***g-effect***) was the dominant positive effect in driving carbon emissions increase, while the energy intensity effect of production (***e-effect-Production***), the economic structure effect (***s-effect***) and the carbon intensity effect of production (***f-effect-Production***) were the main contributing factors suppressing the carbon emission growth at the industrial sector. Considering the high urbanization level in Guangzhou, the affluence effect of urban (***g-effect-AUI***) was the most dominant positive driving factor on the carbon emissions increment, while the energy intensity effect of urban (***e-effect-Urban***) played the most important role in curbing carbon emissions growth at the residential sector.

In conclusion, affluence effect—economic growth, was still the most important contributor to the increases in carbon emissions in urban China. However, structural indicators, such as economic structure and energy intensity of different industrial sector, were playing significant negative effects on carbon emissions. In the future, economic structure adjustment and energy intensity improvement at the detailed production industries should be deeply investigated, in order to make these indicators play more negative effects on carbon emissions.

### Policy recommendations

Considering the significant important role played by the emissions embodied in the imported electricity via the cross-province electricity trades, Guangzhou should adjust its current emissions mitigation policies which only consider its emissions occurring within the city’s boundary. Guangzhou should make more joint efforts to help its neighbors improve the efficiency of thermal power generation. Guangzhou might make efforts to buy more clean power such as hydropower from its neighbors Yunnan and Guangxi provinces to replace a certain amount of thermal power. In addition, carbon trading scheme [87] about the cross-province electricity trades should be introduced and conducted on the imported electricity in Guangzhou.

In order to further promote the negative effects of the economic structure effect (***s-effect***), the energy intensity effect of production (***e-effect-Production***), and the carbon intensity effect of production (***f-effect-Production***), Guangzhou should pay more efforts to the optimization of energy consumption structure and industrial structure at the industrial sector. (1) Coal consumption in energy mix should be further decreased, while relatively low carbon energy such as natural gas should be accelerated both in the industrial and residential sectors. Renewable energy such as solar PV and hydropower should also be encouraged. (2) Efficiency of thermal power generation should be further improved in Guangzhou, especially the major power plants such as the *Pearl River power plant* and the *Huarun power plant* in Nansha district, the *Guangzhou power plant* in Liwan district, the *Hengyun power plant*, the *Huangpu power plant*, and the *Ruiming power plant* in Huangpu district. (3) Energy consumption structure and energy utilization technique should be also effectively optimized and improved, especially in the pillar industries (i. e. *electronic information manufacturing industry*, *automobile industry*, and *petrochemical industry*), the traditional advantage industries (i. e. *iron and steel*, *shipbuilding*, *machinery and equipment*, *paper*, and *textile* etc.) and the new and high technology industries (i. e. *software*, *new material*, *new energy*, and *digital industry* etc.) in Guangzhou in the current and near future.

Urbanization level of Guangzhou is relatively high than other cities in Guangdong province and even in China. The rapid increase in the average income of urban residents along with the high level of urbanization has made the affluence effect of urban (***g-effect-AUI***) become the most dominant positive driving factor on the residential carbon emissions increment during the whole research period. The residential energy consumption structure was mainly based on gasoline, liquefied petroleum gas, and natural gas in Guangzhou. In order to further promote the negative effects of the energy intensity effect of urban (***e-effect-Urban***), more clean fuels such as domestic solar systems should be added to the total residential energy consumption, and low carbon energy consumption behaviors and lifestyles should be advertised and practiced actively.
